# HIV awareness and prevention strategies among transgender women in the Eastern and Southern United States: findings from the LITE Study

**DOI:** 10.1002/jia2.25999

**Published:** 2022-10-12

**Authors:** Rodrigo A. Aguayo‐Romero, Christopher M. Cannon, Andrea L. Wirtz, Erin E. Cooney, Kenneth H. Mayer, Sari L. Reisner, the American Cohort to Study HIV Acquisition among Transgender Women (LITE) Study Group

**Affiliations:** ^1^ Division of Endocrinology, Diabetes, and Hypertension Brigham and Women's Hospital Boston Massachusetts USA; ^2^ The Fenway Institute Fenway Health Boston Massachusetts USA; ^3^ Harvard Medical School Boston Massachusetts USA; ^4^ Research Department Whitman‐Walker Institute Washington DC USA; ^5^ Department of Epidemiology Johns Hopkins Bloomberg School of Public Health Baltimore Maryland USA; ^6^ Department of International Health Johns Hopkins Bloomberg School of Public Health Baltimore Maryland USA; ^7^ Harvard T.H. Chan School of Public Health Boston Massachusetts USA

**Keywords:** transgender women, gender diverse, HIV prevention strategies, latent class analysis, PrEP, PEP

## Abstract

**Introduction:**

Transgender women (TW) experience an increased risk of human immunodeficiency virus (HIV) acquisition. This study identified patterns of HIV awareness and prevention strategies used by TW who were not living with HIV.

**Methods:**

Data were drawn from a baseline survey of the LITE Study, a multi‐site cohort of TW in Eastern and Southern United States (March 2018–August 2020). We conducted a latent class analysis to identify classes of HIV awareness and prevention strategies among TW who reported past 12‐month sexual activity (*N* = 958) using 10 variables spanning HIV knowledge, receipt and use of HIV prevention strategies, and sexual practices. Due to differences across the cohort arms, classes were estimated separately for TW enrolled in site‐based versus online study arms. We identified demographic characteristics, gender‐affirming indicators and HIV vulnerabilities associated with class membership.

**Results:**

Four parallel classes emerged: class 1 “limited strategies—less sexually active” (15% and 9%, site‐based and online, respectively), class 2 “limited strategies—insertive sex” (16%/36%), class 3 “limited strategies—receptive sex” (33%/37%) and class 4 “multiple strategies—insertive and receptive sex” (36%/18%). Across all classes, condomless sex, pre‐exposure prophylaxis (PrEP)/post‐exposure prophylaxis (PEP) prevention knowledge and awareness were high but reported PrEP/PEP use was low. Compared with class 1, membership in class 4 was associated with being a person of colour (site‐based OR = 2.15, 95% CI = 1.15–4.00, online OR = 4.54, 95% CI = 1.09–18.81) increased odds of self‐perceived medium‐to‐high HIV risk (site‐based OR = 4.12, 95% CI = 2.17–7.80, online OR = 11.73, 95% CI = 2.98–46.13), sexually transmitted infections (STI) diagnosis (site‐based OR = 6.69, 95% CI = 3.42–13.10, online OR = 8.46, 95% CI = 1.71–41.78), current sex work (site‐based OR = 6.49, 95% CI = 2.61–16.11, online OR = 10.25, 95% CI = 1.16–90.60) and 2–4 sexual partners in the last 3 months (site‐based OR = 2.61, 95% CI = 1.33–5.13). Class 3, compared with class 1, had increased odds of current sex work partners (site‐based OR = 3.09, 95% CI = 1.19–8.07) and of having 2–4 sexual partners in the last 3 months (site‐based OR = 3.69, 95% CI = 1.85–7.39).

**Conclusions:**

TW have varied HIV awareness and prevention strategy utilization, with clear gaps in the uptake of prevention strategies. Algorithms derived from latent class membership may be used to tailor HIV prevention interventions for different subgroups and those reached through facility‐based or digital methods.

## 1 INTRODUCTION

Human immunodeficiency virus (HIV) disproportionately impacts transgender women (TW). Previous meta‐analyses reflected HIV prevalence estimates ranging from 7% (2012) to 19% (2018) in the United States [1, 2]. The Centers for Disease Control and Prevention (CDC) Report on HIV among TW estimated an HIV prevalence of 42% in seven US cities in 2019–2020 [3]. The CDC report also showed that TW of colour have higher rates of HIV as compared to previous meta‐analyses; 44–62% for Black/African American TW, 26–35% for Hispanic/Latina TW and 7–17% for White TW [2, 3]. Reported behavioural risks underscore HIV prevalence estimates; for example, 38% of TW reported sex work and condomless sex (independently), and 42% reported multiple partnerships [2], as well as dense sexual networks, which facilitate more rapid transmission across networks [4, 5].

Extant literature has documented TW's prevention knowledge and strategies to prevent HIV. Data from a cross‐sectional study in Baltimore and Washington DC demonstrated that TW of colour had high scores of HIV knowledge and HIV risk perceptions [6]. Studies from 2012 to 2020 revealed that three out of four TW got HIV tested within a 12‐month period [2, 3, 6, 7]. A survey among TW in New York City found that TW reported condoms as their first choice (59%), followed by abstinence (14%), pre‐exposure prophylaxis (PrEP) (12%) and limiting the number of partners (9%) [8]. Surveys estimate that 57–63% of TW reported post‐exposure prophylaxis (PEP) knowledge [6, 8], of which 9–13% reported ever using this prevention modality in the past [6, 8]. PrEP use among TW is also limited. This may be due to limited numbers of TW who participated in PrEP clinical trials, concerns of interactions with hormones, side effects and pill burden, and no specific guidelines for PrEP use tailored to the unique experiences of the trans community [9, 10, 11]. Recent research has demonstrated that 87% of TW had PrEP knowledge, and 81% knew how to get it if desired [6]. The CDC report revealed an increase in PrEP awareness (92%); however, PrEP use among TW was limited, with only 32% of HIV uninfected TW reporting PrEP use [3]. HIV prevention requires effective combination strategies to mitigate HIV acquisition risk [12, 13, 14, 15]. Yet, the combinations of HIV prevention strategies TW use to reduce HIV risk and how these may differ for subgroups are unknown.

There is limited research on combination HIV prevention strategies among TW. One review on sex work in TW estimated that the implementation of tailored interventions could decrease the incidence of HIV by 50% in 10 years [15]. More combination prevention strategies for TW are needed [4], but there is no evidence‐based research examining combination HIV prevention strategies utilized individually by TW. This study aimed to fill this gap through latent class analysis (LCA) to explore distinct patterns of HIV prevention strategies among TW in Eastern and Southern United States. These regions have the highest HIV rates across the United States [16]. We sought to determine the association of class membership with demographics, gender‐affirming indicators and HIV vulnerabilities to inform future interventions. This is the first use of LCA among HIV‐negative TW to model combination HIV prevention strategies in the United States.

## 2 METHODS

### 2.1 Data

This study used baseline data from the American Cohort to Study HIV Acquisition among Transgender Women—LITE Study. The LITE cohort included two arms: a technology‐enhanced, site‐based arm (*N* = 732) in six cities in the Eastern and Southern United States (Atlanta, Baltimore, Boston, Miami, New York City and Washington, DC) and an auxiliary online arm that enrolled participants in 72 cities matched on population size and demographics to the cities above (*N* = 582). Participants were enrolled and completed baseline surveys between March 2018 and August 2020. 98.12% (*N* = 940) of participants completed the baseline visit prior to the beginning of the lockdown in the United States on 15 March 2020. TW were recruited via technology‐based (social media and dating apps) and non‐technology‐based (clinical‐based referrals, peer referrals and gender‐affirming events) recruitment methods. Protocols for the LITE Study have been published [17, 18].

Eligibility criteria for participation in the baseline survey included being ≥ age 18 years, reporting a trans feminine identity based on a two‐step measure (being assigned male sex at birth, identifying as woman, female or along the transfeminine spectrum), speaking English or Spanish, a negative HIV test and providing consent to participate in at least the baseline study visit. We restricted this analysis to participants who reported being sexually active (anal or vaginal sex) in the last 12 months regardless of condom use practices (*n* = 577 site‐based arm; *n* = 381 online arm). We were interested in HIV prevention strategies among TW who may be exposed to HIV through condomless sex since this represents a primary mode of HIV acquisition for TW [1]. Study protocols were approved by the Johns Hopkins School of Medicine single institutional review board for all study sites.

### 2.2 Measures

#### 2.2.1 Manifest variables: HIV awareness and prevention strategies

We conceptualized HIV awareness and prevention strategies as a combination of 10 psychoeducational, biomedical use (PEP [19, 20] and PrEP [21, 22, 23]), and behavioural interventions and strategies carrying varying degrees of HIV risk and protective levels. For instance, we included oral sex as a strategic behaviour with a significantly lower risk of HIV acquisition than engaging in anal and/or vaginal sex. Table 1 contains a detailed description of the manifest variables.

**Table 1:** Measures for manifest variables included in latent class analysis models in the LITE Study of transgender women in the United States (*N* = 958)

Variable	Measure description
HIV information from organizations	Based on answering “Yes” to any of the three following responses to the question “In the last 3 months, have you received any of the following services from a clinic, community organization or health facility (other than in this study)?”: “One‐on‐one conversation with an outreach worker, counsellor or prevention programme worker;” “Participated in an organized group session to discuss ways to prevent HIV infections;” or “Received HIV/STI prevention information (e.g. a flyer or info sheet)”
HIV knowledge	Based on answering the following two questions correctly: “What type of sex puts someone most at risk for HIV infection?” (answer = anal) and “Can someone get HIV from sharing a needle to inject hormones or silicone?” (answer = yes)
PrEP/PEP awareness	Based on answering “Yes” to either of the following questions: “Have you ever heard about PrEP (pre‐exposure prophylaxis) for the prevention of HIV infection in people who are HIV‐negative?” or “Have you heard of PEP for preventing HIV after someone has had possible contact with HIV (e.g. after unsafe sex or rape)?”
HIV test last year	Based on selecting any of the three response options to the question: “When was your most recent HIV test? If you're not sure, please give your best guess. If you are living with HIV, this refers to when you were first told that you have HIV.” Responses: “Less than 3 months ago,” “3–6 months ago,” or “7–11 months ago.”
PrEP use ever	Based on answering “Yes” to the question: “Have you ever taken PrEP (pre‐exposure prophylaxis) for the prevention of HIV infection?”
PEP use ever	Based on answering “Yes” to the question: “Have you ever taken PEP?”
Condomless sex	Based on answering “Yes” to the question: “In the last 12 months, have you ever had sex (anal or vaginal) without a condom?”
Receptive anal/vaginal sex	Based on answering “Yes” to any of the two sexual behaviours on the question: “Which type(s) of sex did you have with (casual partners, regular partners or sex work clients) in the last 3 months?” Behaviours: “Receptive anal sex (a partner put their penis in your anus or butt)” or “Receptive vaginal sex (a partner put their penis into your vagina).”
Insertive anal/vaginal sex	Based on answering “Yes” to any of the two sexual behaviours on the question: “Which type(s) of sex did you have with (casual partners, regular partners or sex work clients) in the last 3 months?” Behaviours: “Insertive anal sex (you put your penis in a partner's anus or butt)” or “Insertive vaginal sex (you put your penis in a partner's vagina.).”
Oral sex	Based on answering “Yes” to any of the four sexual behaviours on the question: “Which type(s) of sex did you have with (casual partners, regular partners or sex work clients) in the last 3 months?” Behaviours: “Receptive penile oral sex (a partner put their penis in your mouth),” “Insertive penile oral sex (you put your penis in a partner's mouth),” “Received oral‐vaginal sex (a partner put their mouth on your vagina),” or “Performed oral‐vaginal sex (you put your mouth on a partner's vagina).”

*Note*. All manifest variables were recoded as binary (yes vs. no).

Abbreviations: PEP, post‐exposure prophylaxis; PrEP, pre‐exposure prophylaxis; STI, sexually transmitted infection.

#### 2.2.2 Covariates

Demographics, gender‐affirming variables and HIV vulnerabilities were used as covariates and were selected based on documented relationships with HIV prevention strategies in the transgender HIV literature. Table S1 includes detailed covariate descriptions.

### 2.3 Statistical analysis

LCA was selected to empirically identify distinct classes of HIV awareness and prevention strategies. LCA is a person‐centred statistical approach of identifying underlying patterns or subgroups—also known as latent classes—sharing similar characteristics based on the interconnectedness of multiple observed categorical variables [24, 26]. Previous LCA research has focused on TW living with HIV and HIV‐related health outcomes but not on their prevention strategies [26, 27, 28].

All analyses were conducted using SAS 9.4. Data were analysed using complete cases since missing data was 0.10% and only for HIV risk and social support. Where data were missing due to skip patterns, we specified that participants did not receive the question(s). For instance, HIV risk includes a category for those who noted they never received an HIV test and, therefore, did not receive the subsequent question about HIV risk. We tested measurement invariance between site‐based and online arms. We used PROC LCA 1.3.2 macro [29, 30] to identify the LCA baseline model, which refers to the base model that does not include grouping variables or covariates. The determination of the base models for each arm was based on multiple indicators, including maximum likelihood solution percentage, Akaike's information criterion, Bayesian information criterion, a bootstrap likelihood ratio test and entropy (Table S2) [29]. Lastly, we used a three‐step covariates macro in SAS to estimate the odds ratios statistically predicting class membership from the covariates [31, 32].

## 3 RESULTS

### 3.1 Study population

Half of TW in both arms were 18–29 years old (58%), had some college education or higher (70%), had incomes above the federal poverty line (FPL) (54%) and lived in the North (51%; Table 2).

**Table 2:** Social demographics of sexually active transgender women in the LITE Study, Eastern and Southern United States (*N* = 958)

Characteristics		Site‐based (*n* = 577) *n* (%)	Online (*n* = 381) *n* (%)	Total (*N* = 958) *n* (%)
Age in years	18–29	320 (56)	231 (61)	551 (58)
	30–39	147 (25)	106 (28)	253 (26)
	40+	110 (19)	44 (11)	154 (16)
Race/ethnicity	Non‐Hispanic White	186 (32)	287 (75)	473 (49)
	Non‐Hispanic Black	122 (21)	17 (4)	139 (15)
	Hispanic White	58 (10)	12 (3)	70 (7)
	Hispanic Black	21 (4)	1 (0.3)	22 (2)
	Non‐Hispanic and more than one race or other	83 (14)	47 (12)	130 (14)
	Hispanic and more than one race or other	98 (17)	15 (4)	113 (12)
	Unknown	9 (2)	2 (0.5)	11 (1)
Education	<HS Diploma/GED	212 (37)	70 (18)	282 (29)
	≥ Some college	360 (62)	309 (81)	669 (70)
	Unknown	5 (0.9)	2 (0.5)	7 (0.7)
Income	Above FPL	263 (46)	255 (67)	518 (54)
	Below FPL	223 (39)	87 (23)	310 (32)
	Unknown	91 (16)	39 (10)	130 (14)
Employment	Full‐time	174 (30)	181 (48)	355 (37)
	Part‐time	139 (24)	80 (21)	219 (23)
	Not employed	250 (43)	108 (28)	358 (37)
	Unknown	14 (2)	12 (3)	26 (3)
Insurance	Uninsured	63 (11)	37 (10)	100 (10)
	Public	279 (48)	90 (24)	369 (39)
	Private	192 (33)	234 (61)	426 (45)
	Unknown	43 (7)	20 (5)	63 (7)
Region	North	296 (51)	189 (50)	485 (51)
	Mid‐Atlantic	151 (26)	64 (17)	215 (22)
	South	130 (23)	128 (33)	258 (27)

Abbreviations: FPL, federal poverty line; GED, general educational development; HS, high school.

Half of the participants identified as trans woman/trans female (51%), though gender identities varied (Table S3). Nearly, half (47%) had at least one gender‐affirming procedure and a majority of TW in both arms reported being on hormone therapy. Nearly, three‐quarters of the sample said they had not received HIV prevention education or trans‐specific materials in the last 3 months (72%). More than half of TW reported low social support in the past 6 months.

Among all participants, vulnerabilities included never testing for STI (22%), lifetime involvement in sex work (42%), two or more sexual partners in the past 12 months (56%) and 27% medium to high perceived risk for HIV acquisition (Table S4). Sixty‐four percent reported partnerships with cisgender men, though the genders of sexual partners were diverse.

### 3.2 Four class model

**Figure 1:**
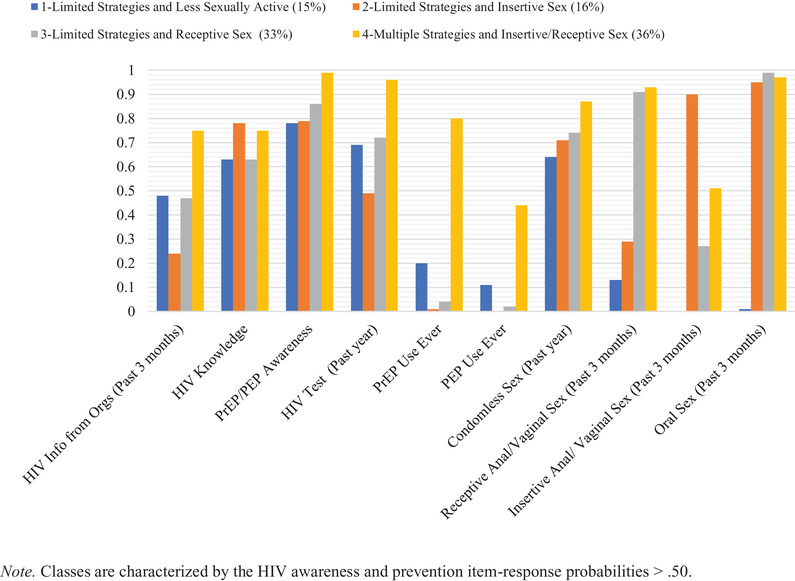
Probability of endorsing each HIV awareness and prevention item among transgender women in the LITE Study, Eastern and Southern United States—site‐based (*N* = 577). Abbreviations: Orgs, organizations; PEP, post‐exposure prophylaxis; PrEP, pre‐exposure prophylaxis.

**Figure 2:**
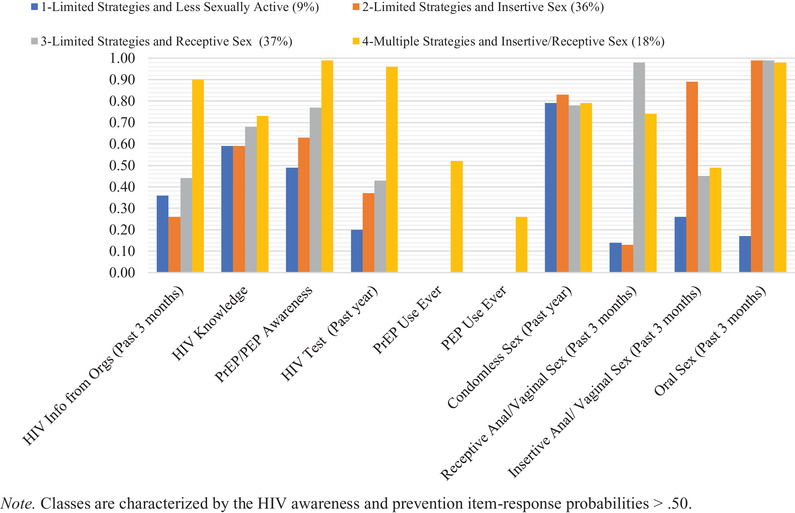
Probability of endorsing each HIV awareness and prevention item among transgender women in the LITE Study, Eastern and Southern United States—online (*N* = 381). Abbreviations: Orgs, organizations; PEP, post‐exposure prophylaxis; PrEP, pre‐exposure prophylaxis.

The *G* ^2^ difference test (*G* ^2^ = 718.08, *df* = 44, *p<*0.001) was statistically significant, suggesting that measurement invariance did not hold across site‐based and online arms. Therefore, we conducted separate analyses for the two arms. Five competing models containing 10 manifest variables were compared to determine a well‐identified LCA baseline model in both arms. The model with four classes for the site‐based arm (*G* ^2^ of 416.52 with 980 degrees of freedom) and the online arm (*G* ^2^ of 302.78 with 980 degrees of freedom) were selected as final models because these provided an optimal balance between statistical criteria and interpretability. Classes in both models had similar characteristics but differed in class prevalence and probability of particular strategies. Given both models’ parallel nature, we used the same class names for both arms. The four classes in each arm were labelled as: class 1—limited strategies and less sexually active, class 2—limited strategies and insertive sex, class 3—limited strategies and receptive sex and class 4—multiple strategies and insertive/receptive sex. The composition of classes in site‐based and online arms can be seen in Figures 1 and 2 (see Tables S5 and S6 for item‐response probabilities and prevalence of awareness and prevention strategies). Analysis was restricted to those who were sexually active in the last 12 months. Yet, class 1 emerged to represent TW who had a very low probability of having had sex in the previous 3 months before the survey. Class 4 was the largest group (36%) in the site‐based arm, characterized by high probabilities of 9 out of the 10 HIV awareness and prevention strategies (all except for prior PEP use). Class 3 was the largest (37%) in the online arm, characterized by high probabilities of HIV knowledge, PrEP/PEP awareness, condomless sex, receptive anal/vaginal sex and oral sex.

The classes in both arms were differentiated by self‐reported behaviours. All site‐based classes had a high probability of HIV testing (range 0.49–0.96), while it was only a characteristic of class 4 in the online cohort. Participants in both arms and across all classes exhibited high probabilities of being knowledgeable about HIV, PrEP, and PEP and engaging in condomless sex in the past year. The probability of PrEP/PEP awareness was just below the 0.50 threshold in the online class 1. All site‐based and online classes were characterized by a low probability of lifetime PEP use (18% site‐based arm; 5% online arm; see Tables S5 and S6). PrEP use was only a characteristic of class 4 in both arms.

#### 3.2.1 Demographic covariates

As seen in Table 3, people of colour had higher odds of belonging to class 4 in both arms. Similarly, TW with ≥ some college education were more likely to be in class 2 in both arms. In the site‐based cohort, TW with private insurance had higher odds of belonging to class 2. In the online cohort, TW living below the FPL and with public insurance had higher odds of belonging to class 4.

**Table 3:** Covariates table: demographics associated with class membership in site‐based and online cohorts among transgender women in the LITE Study, Eastern and Southern United States (March 2018–August 2020)

	Site‐based arm (*N* = 577)			Online arm (*N* = 381)		
Class	2	3	4	2	3	4
	Limited strategies and insertive sex	Limited strategies and receptive sex	Multiple strategies and insertive/receptive sex	Limited strategies and insertive sex	Limited strategies and receptive sex	Multiple strategies and insertive/receptive sex
Age in years (continuous)	1.32 (0.90–1.93)	1.36 (0.99–1.87)	1.24 (0.93–1.66)	**0.55 (0.37**–**0.82)**	**0.62 (0.44**–**0.88)**	0.73 (0.52–1.02)
POC (reference: White)^a^	**0.11 (0.04**–**0.26)**	1.11 (0.59–2.08)	**2.15 (1.15**–**4.00)**	2.11 (0.52–8.50)	3.79 (1.00–14.35)	**4.54 (1.09**–**18.81)**
≥ Some college (reference: HS Diploma GED)	**8.85 (2.73**–**28.68)**	1.16 (0.66–2.05)	1.23 (0.72–2.10)	**3.96 (1.20**–**13.11)**	1.49 (0.57–3.90)	0.76 (0.26–2.17)
Income below federal poverty level (reference: above)	**0.44 (0.21**–**0.89)**	**0.43 (0.24**–**0.78)**	0.80 (0.47–1.37)	1.12 (0.31–4.03)	2.16 (0.66–7.08)	**4.38 (1.24**–**15.44)**
Employment						
Employed part‐time	1.94 (0.86–4.38)	1.76 (0.87–3.59)	1.38 (0.69–2.75)	1.68 (0.49–5.78)	2.46 (0.76–7.99)	1.06 (0.26–4.37)
Employed full‐time	1.41 (0.69–2.90)	1.09 (0.58–2.02)	0.91 (0.50–1.64)	**0.29 (0.11**–**0.80)**	**0.23 (0.09**–**0.60)**	**0.15 (0.05**–**0.46)**
Insurance						
Uninsured	0.85 (0.26–2.77)	1.65 (0.70–3.87)	0.66 (0.26–1.69)	0.63 (0.17–2.41)	0.69 (0.20–2.45)	0.66 (0.15–2.99)
Public	**0.41 (0.20**–**0.85)**	0.69 (0.39–1.22)	1.50 (0.87–2.57)	1.02 (0.32–3.29)	1.39 (0.46–4.22)	**4.07 (1.26**–**13.14)**
Private	**3.00 (1.48**–**6.10)**	1.06 (0.57–1.95)	0.68 (0.37–1.23)	1.16 (0.44–3.01)	0.59 (0.24–1.45)	**0.30 (0.11**–**0.84)**
Region						
North	1.71 (0.85–3.44)	0.79 (0.45–1.40)	0.99 (0.58–1.69)	1.27 (0.53–3.02)	1.11 (0.48–2.57)	1.00 (0.38–2.61)
Mid‐Atlantic	0.49 (0.20–1.17)	0.91 (0.48–1.72)	1.11 (0.62–2.00)	0.72 (0.22–2.42)	0.82 (0.26–2.59)	2.19 (0.67–7.22)
South	0.95 (0.40–2.22)	1.51 (0.78–2.93)	0.89 (0.45–1.72)	0.92 (0.37–2.26)	1.00 (0.42–2.38)	0.51 (0.18–1.48)

*Note*. Limited strategies and less sexually active is the reference group. Odds ratios are unadjusted. Boldface indicates statistically significant association—CI does not contain 1.0. Unknown responses were included in the modelling.

^a^ People of Colour (POC) include every race/ethnicity other than non‐Hispanic White versus non‐Hispanic White. Recoded because of the low prevalence of some racial groups. Unemployed did not yield robust estimates and was not included in this table.

#### 3.2.2 Gender‐affirming covariates

TW receiving trans‐specific HIV prevention information in the past 3 months had higher odds of being in class 4 and TW who had high social support had higher odds of being in class 2 in both arms (Table 4). The remaining covariates were only associated with membership in class 2. TW who identified as non‐binary (NB) or another gender diverse identity and who were currently taking hormones had higher odds of belonging to class 2. While those TW who identified as woman/female had lower odds of belonging to class 2 than those in class 1.

**Table 4:** Covariates table: gender‐affirming variables associated with class membership in site‐based and online arms among transgender women in the LITE Study, Eastern and Southern United States (March 2018–August 2020)

	Site‐based arm (*N* = 577)			Online arm (*N* = 381)		
	2	3	4	2	3	4
Class	Limited strategies and insertive sex	Limited strategies and receptive sex	Multiple strategies and insertive/receptive sex	Limited strategies and insertive sex	Limited strategies and receptive sex	Multiple strategies and insertive/receptive sex
Gender identity						
Woman/female	**0.44** **(0.20**–**0.96)**	0.99 (0.56–1.76)	0.85 (0.49–1.46)	1.22 (0.39–3.82)	1.58 (0.53–4.69)	0.65 (0.16–2.61)
Transwoman/transfemale	1.20 (0.60–2.38)	0.85 (0.48–1.50)	0.85 (0.50–1.46)	1.16 (0.45–2.99)	0.71 (0.29–1.73)	1.11 (0.39–3.21)
Non‐binary or other gender diverse^a^	**3.81** **(1.18**–**12.29)**	1.86 (0.58–5.98)	2.64 (0.90–7.70)	0.53 (0.13–2.12)	1.04 (0.31–3.50)	1.26 (0.32–4.91)
Hormone therapy (past 3 months)	**3.55** **(1.02**–**12.28)**	1.10 (0.55–2.18)	1.35 (0.69–2.66)	1.27 (0.49–3.30)	0.89 (0.36–2.20)	2.26 (0.70–7.26)
Any gender‐affirming procedure	1.44 (0.73–2.84)	1.02 (0.58–1.80)	1.54 (0.90–2.63)	0.65 (0.27–1.57)	0.67 (0.28–1.57)	2.25 (0.84–6.01)
Trans‐specific HIV prevention info	0.09 (0.01–1.03)	1.20 (0.63–2.29)	**1.95** **(1.07**–**3.55)**	0.49 (0.11–2.09)	0.97 (0.28–3.43)	**4.78** **(1.37**–**16.64)**
Social support (high vs. low)	**3.17** **(1.56**–**6.45)**	1.75 (0.97–3.16)	1.42 (0.81–2.49)	**2.47** **(1.01**–**6.05)**	1.20 (0.50–2.88)	1.53 (0.57–4.12)

*Note*. Limited strategies and less sexually active is the reference group. Odds ratios are unadjusted. Boldface indicates significant association—CI does not contain 1.0. “Prefer not to answer” and “Don't know” responses were included in the modelling.

^a^ Includes non‐binary, woman of trans experience, person of trans experience, two‐spirit and other identities.

#### 3.2.3 HIV vulnerabilities covariates

Participants in both arms self‐reporting a positive sexually transmitted infection (STI) test, sex work and medium‐to‐high HIV risk level had higher odds of being in class 4 (Table 5). Current sex work was also associated with membership in class 3 for the site‐based arm. Significant associations between the number of partners and class membership emerged for the site‐based arm. TW who reported one partner in the last 3 months had higher odds of belonging to class 2, while those with 2–4 partners had higher odds of being in classes 3 and 4. TW in the site‐based arm who reported partnerships with cisgender women (CW), transgender men (TM) and non‐binary (female at birth—NB FAB) partners had higher odds of belonging to class 2. Participants who reported cisgender men (CM) as partners had higher odds of being in site‐based classes 3 and 4, and those who reported TW and NB (male at birth—MAB) partners had higher odds of being in class 3 for both arms and in the online class 4.

**Table 5:** Covariates table: HIV vulnerabilities associated with class membership in site‐based and online arms among transgender women in the LITE Study, Eastern and Southern United States (March 2018–August 2020)

	Site‐based arm (*N* = 577)			Online arm (*N* = 381)		
Class	2	3	4	2	3	4
	Limited strategies and insertive sex	Limited strategies and receptive sex	Multiple strategies and insertive/receptive sex	Limited strategies and insertive sex	Limited strategies and receptive sex	Multiple strategies and insertive/receptive sex
Positive STI test result (vs. negative‐lifetime)	0.37 (0.10–1.42)	1.70 (0.82–3.52)	**6.69** **(3.42**–**13.10)**	0.12 (0.00–11.33)	1.34 (0.25–7.13)	**8.46** **(1.71**–**41.78)**
Sex work (lifetime)	0.47 (0.22–1.02)	1.18 (0.67–2.10)	**3.37** **(1.93**–**5.89)**	0.72 (0.23–2.22)	2.10 (0.77–5.78)	**4.29** **(1.42**–**13.02)**
Sex work (current)	0.29 (0.02–3.97)	**3.09** **(1.19**–**8.07)**	**6.49** **(2.61**–**16.11)**	0.46 (0.02–11.04)	4.21 (0.49–36.18)	**10.25** ^a^ **(1.16**–**90.60)**
HIV risk						
Med to high risk	0.52 (0.18–1.51)	1.67 (0.84–3.32)	**4.12** **(2.17**–**7.80)**	0.10 (0.00–6.77)	2.39 (0.64–8.96)	**11.73** **(2.98**–**46.13)**
Low risk	0.80 (0.40–1.59)	1.05 (0.59–1.85)	0.64 0.37–1.10)	1.66 (0.61–4.52)	1.68 (0.64–4.45)	1.61 (0.54–4.84)
No risk	0.84 (0.34–2.09)	0.65 (0.29–1.45)	0.66 (0.31–1.37)	0.88 (0.32–2.46)	0.39 (0.13–1.17)	0.42 (0.11–1.56)
No. of sex partners						
One partner	**7.75** **(3.52**–**17.07)**	1.95 (0.98–3.85)	0.88 (0.44–1.76)	5.22 (2.00–13.61)	0.55 (0.23–1.31)	0.29 (0.10–0.88)
2–4 partners	2.04 (0.89–4.66)	**3.69** **(1.85**–**7.39)**	**2.61** **(1.33**–**5.13)**	–	–	–
Gender of sex partners						
(12 months)						
Cisgender men	**0.05** **(0.02**–**0.15)**	**3.55** **(1.23**–**10.22)**	**6.34** **(2.59**–**15.50)**	–	–	–
Cisgender women	**18.45** **(7.29**–**46.71)**	0.73 (0.29–1.83)	0.91 (0.44–1.92)	1.75 (0.70–4.35)	**0.24** **(0.10**–**0.58)**	0.53 (0.20–1.40)
TW/NB (MAB)	2.22 (0.97–5.08)	**2.27** **(1.11**–**4.66)**	1.50 (0.74–3.05)	3.31 (0.61–17.91)	**18.99** ^a^ **(3.76**–**95.84)**	**13.23** ^a^ **(2.45**–**71.43)**
TM/NB (FAB)	**5.68** **(2.45**–**13.17)**	0.98 (0.40–2.42)	0.91 (0.40–2.06)	1.83 (0.64–5.26)	0.87 (0.29–2.58)	2.20 (0.71–6.86)

*Note*. Limited strategies and less sexually active is the reference group. Odds ratios are unadjusted. Boldface indicates significant association—CI does not contain 1.0. Positive STI test—last 3 months, zero partners, 2–4 partners (online arm only), 5+ partners and cisgender men (online arm only) did not yield robust estimates and were not included in this table. “Prefer not to answer” responses and never tested category were also included in the model when applicable. HIV risk is missing data from one participant.

^a^ Unstable confidence intervals should be interpreted with caution.

Abbreviations: FAB, female assigned at birth; MAB, male assigned at birth; NB, non‐binary; STI, sexually transmitted infection; TM, transgender men; TW, transgender women.

## 4 DISCUSSION

We identified four classes of combination HIV awareness and prevention strategies being used by TW and evaluated characteristics associated with using these strategies. We identified gaps where increased HIV prevention efforts should be allocated. Consistent with the literature on HIV vulnerabilities [1, 2, 4, 6, 7], TW in classes 3—limited strategies and receptive sex and 4—multiple strategies and insertive/receptive sex in both arms were at increased HIV risk due to engaging in condomless sex and receptive sex, which was associated with higher odds of STI diagnosis, sex work and multiple partners within a 3‐month period. TW of colour in class 4—multiple strategies and insertive/receptive sex were at heightened risk for HIV at the intersection of gender and race/ethnicity. The association of self‐assessed medium‐to‐high HIV risk perception, STI history and sex work with class 4—multiple strategies and insertive/receptive sex membership could indicate that the multiple strategies utilized are an adaptive response to previous experiences, indicating greater resilience when facing high HIV vulnerability. In contrast, class 3—limited strategies and receptive sex was also at heightened risk but only utilizes a limited number of strategies.

Meanwhile, classes 1—limited strategies and less sexually active and 2—limited strategies and insertive sex in both arms had profiles indicating lower HIV risk attributed to either low probabilities of sexual activity in the last 3 months or a high probability of insertive sexual positioning. Covariates associated with class 2—limited strategies and insertive sex included having ≥ some college education, private insurance, lower odds of income below the FPL, increased social support and a lower number of partners who were predominantly CW, TM or NB (FAB) individuals, which have all been identified as protective or mitigating factors in the literature [4, 33–36]. These findings demonstrate that partnerships are diverse among TW and not universally with CM. This suggests that tailored HIV prevention programming is needed to recognize and discuss appropriate strategies across various partnerships and sexual practices.

PrEP and PEP awareness was high in almost all classes for both arms, consistent with previous studies [9, 11, 37]. But awareness did not appear to reflect PrEP or PEP uptake overall. This could be due to limited points of access to biomedical interventions, especially outside of larger urban centres. Class 4—multiple strategies and insertive/receptive sex in both arms was characterized by PrEP use and information from organizations, while no classes were characterized by PEP use. The larger size of the site‐based class 4—multiple strategies and insertive/receptive sex and higher probability of PrEP use compared to the online equivalent may be due to the availability of medical care and services that TW in the site‐based arm have access to. Low PEP uptake is likely due to limited access and the 72‐hour time window needed to start treatment. Meanwhile, HIV testing was a prevention strategy underutilized in the online arm. Online classes 1–3 showed that TW are not regularly tested. High HIV testing utilization in the four site‐based classes (77%) compared to the online arm (48%) might be attributed to their direct linkage and engagement with health or social services organizations. Overall, TW face an array of barriers that hinder their access and uptake of prevention strategies that include but are not limited to insurance coverage, education, transportation, education, stigma, discrimination and low HIV perception risk [37, 38].

### 4.1 Implications

A common pattern across all classes was the high probability of having engaged in condomless sex in the past 12 months, indicating that efforts should be redirected towards other prevention strategies. Therefore, current educational outreach efforts, which have led TW to be knowledgeable about HIV, PrEP and PEP, should expand their reach to the greater TW community and need improved efforts to link TW with these prevention strategies. Health educators may need to look for newer methods and/or novel media tools to better reach the population. The private sector, such as television and film, could help reach more people by getting more HIV storylines into mainstream media.

Class 4—multiple strategies and insertive/receptive sex demonstrated that using multiple prevention strategies is possible, but many of these require access to structural supports for their uptake. Structural supports, like health and social services organizations, need to explore alternative schedules, telehealth, mobile healthcare and/or mail delivery services. TW communities are often unable to and/or uncomfortable accessing physical facilities. Providers need to make their facilities more trans‐inclusive to ensure patients are respected, affirmed and welcomed. Online arm findings highlight the need for public health departments and providers to increase engagement with online promotion methods and outreach to increase PrEP and PEP uptake. Online medical services may increase access for many TW across the country [38, 39]. Providers need to do more in‐person education during all medical appointments; however, increasing accurate and easy‐to‐understand educational opportunities online about biomedical prevention options may be necessary to reach TW who are in increased need and not affiliated with a medical clinic. Given the significant differences found among TW in online versus site‐based arms, different intervention models may be required to fill prevention gaps among TW. Overall, increasing telehealth options may fill some of those gaps, but it will be important to be thoughtful about TW who may not be reached due to lack of technology access or other barriers.

Except for online class 4—multiple strategies and insertive/receptive sex, TW in the other online classes did not get HIV tested the year before the study. Given that HIV self‐testing has gained acceptability among TW in the United States [40], TW in online classes 2—limited strategies and insertive sex and 3—limited strategies and receptive sex may benefit from free or low‐cost home self‐testing HIV kits and more mobile testing clinics or events. Meanwhile, online class 1—limited strategies and less sexually active may not need to participate in HIV testing as frequently. PrEP and PEP awareness was high among all classes except online class 1. However, class 4—multiple strategies and insertive/receptive sex was characterized by PrEP use. Although all PrEP indicated participants could benefit from PrEP programmes that include in‐person and telehealth options, TW in class 3—limited strategies and receptive sex for both arms would benefit from PrEP referrals by healthcare providers. Further, TW in online class 3 may be more likely to uptake PrEP through programmes focused on individual needs or preferences, such as telehealth services and home delivery to maximize accessibility. In contrast, TW in site‐based class 3 should be linked to local PrEP services, particularly if already receiving care from local health centres. Participants across classes may benefit from the availability of long‐acting injectable PrEP, approved by the United States Food and Drug Administration (FDA) in 2021 [41], which has the potential to address some of the barriers associated with oral PrEP, such as adherence. The probability of PEP use was low among all classes in both arms. More research is needed to understand PEP uptake/decision‐making when indicated, given that increasing PEP access could increase opportunities to link and engage TW in PrEP care. Moreover, our results highlight that LCA is a tool that can inform providers and public health departments in HIV prevention efforts with TW. These findings can guide where they allocate prevention resources based on the set of manifest variables that we used to conduct the analysis. Primarily, we have identified that the manifest variables endorsed by TW in classes 3—limited strategies and receptive sex and 4—multiple strategies and insertive/receptive sex make them more vulnerable to HIV infection and should be prioritized.

One major study limitation is that the three‐step covariates macro produced robust estimates when including one covariate at a time, which could be attributed to our sample size and multiple manifest variables. Adding multiple covariates of the same category as in the case of employment and region did not converge. The inability to estimate multivariable models could have led to confounding effects. Studies with larger samples, particularly with online arms, are needed to estimate adjusted multivariable models. Although multi‐site studies have greater generalizability than single‐site studies, data of this subsample are not representative since we restricted inclusion to sexually active TW.

## 5 CONCLUSIONS

Our findings demonstrate that sexually active TW in the Eastern and Southern United States are characterized by four distinct classes of HIV awareness and prevention strategies associated with different levels of vulnerability to HIV. Findings indicated that prevention efforts should prioritize combination strategies among TW, with a particular focus on HIV testing and PrEP. Future interventions may use algorithms derived from latent classes to target TW reached in‐person or online. Honouring and acknowledging the steps TW currently take to prevent HIV and offering tailored support and services to meet HIV prevention goals will be important moving forward.

## COMPETING INTERESTS

The authors report no competing interests.

## AUTHORS’ CONTRIBUTIONS

The following are members of the collaborative author, American Cohort to Study HIV Acquisition Among TW (LITE): Sari L. Reisner (multiple PI, Harvard University, BWH); Andrea L. Wirtz (multiple PI; JHU); Keri Althoff (JHU); Chris Beyer (JHU); James Case (JHU); Erin E. Cooney (JHU); Oliver Laeyendecker (JHU); Dee Adams (JHU); Megan Stevenson (JHU); Tonia Poteat (University of North Carolina); Kenneth H. Mayer (Fenway Health); Asa Radix (Callen‐Lorde Community Health Center); Christopher M. Cannon (Whitman‐Walker Institute); Jason Schneider (Emory University and Grady Hospital); J. Sonya Haw (Emory University and Grady Hospital); Allan Rodriguez (University of Miami); Andrew Wawrzyniak (University of Miami); and the LITE Community Advisory Board, including the following individuals: Flora Marques, Sherri Meeks, Sydney Shackelford, Nala Toussaint, SaVanna Wanzer, and, as well as those who have remained anonymous. ALW and SLR conceptualized and oversaw the LITE site‐based and online arms; RAAR and CMC conceptualized this analysis and wrote the first draft of the manuscript; RAAR analyzed the data. RAAR, CMC, EEE, ALW, KHM, and SLR reviewed, edited, and approved the final manuscript.

## FUNDING

Research reported in this publication was jointly supported by the National Institute of Allergy and Infectious Diseases, the National Institute of Mental Health and the National Institute of Child Health and Human Development of the National Institutes of Health under Award Number UG3/UH3AI133669 (ALW and SLR). Research reported in this publication was also supported by HIV/AIDS, Hepatitis, STD and TB Administration (HAHSTA), Washington, DC, Department of Health. A diversity supplement supports RAAR from the National Institute of Allergy and Infectious Diseases of the National Institutes of Health under Award Number (UH3AI133669‐S2). EEC is supported by a predoctoral fellowship from the National Institute of Mental Health (F31MH124582).

## DISCLAIMER

The content is solely the authors’ responsibility and does not necessarily represent the official views of the National Institutes of Health or HAHSTA.

## Supporting information


**Table S1**. Measures for covariates included in latent class analysis models in the LITE Study of transgender women in the Eastern and Southern United States (*N* = 958).
**Table S2**. Goodness‐of‐fit criteria for competing latent class models among transgender women in the LITE Study, Eastern and Southern United States (*N* = 958).
**Table S3**. Gender‐affirming characteristics of sexually active transgender women in the LITE Study, Eastern and Southern United States (*N* = 958).
**Table S4**. HIV vulnerabilities of sexually active transgender women in the LITE Study, Eastern and Southern United States (*N* = 958).
**Table S5**. Item‐response probabilities for four‐class model: the probability of endorsing each HIV awareness and prevention item‐site based among transgender women in the LITE Study, Eastern and Southern United States (*N* =  577).
**Table S6**. Item‐response probabilities for four‐class model: the probability of endorsing each HIV awareness and prevention item online among transgender women in the LITE Study, Eastern and Southern United States (*N* =  381).

## Data Availability

The authors confirm that all data underlying the findings are fully available upon request. Requests should be sent to the PIs, sreisner@bwh.harvard.edu and awirtz1@jhu.edu.
