# Supplementary material for: HIV awareness and prevention strategies among transgender women in the Eastern and Southern United States: findings from the LITE Study
Source: J Int AIDS Soc. 2022 Oct 12;25(Suppl 5):e25999. doi: 10.1002/jia2.25999 (PMC9557018; doi:10.1002/jia2.25999)
Supplement: Supplementary file 1 — Table S1. Measures for covariates included in latent class analysis models in the LITE Study of transgender women in the Eastern and Southern United States (N = 958). Table S2. Goodness‐of‐fit criteria for competing latent class models among transgender women in the LITE Study, Eastern and Southern United States (N = 958). Table S3. Gender‐affirming characteristics of sexually active transgender women in the LITE Study, Eastern and Southern United States (N = 958). Table S4. HIV vulnerabilities of sexually active transgender women in the LITE Study, Eastern and Southern United States (N = 958). Table S5. Item‐response probabilities for four‐class model: the probability of endorsing each HIV awareness and prevention item‐site based among transgender women in the LITE Study, Eastern and Southern United States (N = 577). Table S6. Item‐response probabilities for four‐class model: the probability of endorsing each HIV awareness and prevention item online among transgender women in the LITE Study, Eastern and Southern United States (N = 381). [file JIA2-25-e25999-s001.docx]

| **Table S1. Measures for Covariates Included in Latent Class Analysis Models in the LITE Study of Transgender Women in the Eastern and Southern U.S. (N=958)** | |
| --- | --- |
| **Variable** | **Measure description** |
| **Social Demographics** |  |
| Age | Based on the question “What is your age as of today?” Categorical for a descriptive table; three categories: 18-29, 30-39, or 40+. Continuous; for covariate analysis |
| Race/Ethnicity | Categorical; seven categories based on the question “Which of the following describes your race?”: Non-Hispanic White, Non-Hispanic Black, Hispanic White, Hispanic Black, Non-Hispanic and more than one race or other, Hispanic and more than one race or other, or Unknown. Binary for covariate analysis: People of color or Non-Hispanic White |
| Education | Categorical; three categories: HS Diploma/GED or less, College or higher, or Unknown |
| Income | Categorical; three categories: Above FPL, Below FPL, or Unknown. Derived from participant’s household income in the past 30 days and FPL |
| Employment | Categorical; four categories: Full Time, Part Time, Not Employed, or Unknown |
| Insurance | Categorical; four categories: Uninsured, Public, Private, or Unknown. Derived from collapsing a current health insurance response |
| Region | Categorical; three categories: North, Mid-Atlantic, or South. Derived from the city of residence |
| **Gender-Affirming** |  |
| Gender Identity | Categorical; eight categories: ‘Woman, Female,’ ‘Transwoman, Transfemale,’ ‘Transfeminine, Transfemme,’ ‘Non-Binary (agender, gender fluid, gender variant, gender non-conforming),’ ‘Woman of trans experience,’ ‘Person of trans experience,’ ‘Transgender women-Two Spirit,’ or ‘Other Identity.’ |
| Hormone Therapy  (Past 3 months) | Based on the question, “In the last 3 months, have you taken hormones for your gender identity or gender transition?” Categorical; three categories: ‘Yes, ‘No,’ or ‘Prefer not to answer.’ |
| Any Gender  Affirming  Procedure | Binary, based on selecting ‘Have had it’ to any gender transition procedures and health care done by a licensed medical professional: Categorical; five categories: ‘Hair removal/electrolysis, breast augmentation/surgery, orchiectomy/removal of testes, vaginoplasty/bottom surgery, or facial feminization surgery (such as nose, brow, chin, cheeks). |
| Trans-specific  HIV Prev. Info.  (Past 3 months) | Categorical; four categories: ‘Yes,’ ‘No,’ ‘Prefer not to answer,’ or ‘Don’t know.’ Based on the question: “In the past 3 months, have you received information on the prevention of HIV or STI tailored specifically for transgender people?” |
| Social Support  (Past 6 months) | Categorical; three categories: ‘High >12,’ ‘Low <=12,’ or ‘Prefer not to answer.’ Derived from the sum of 5 items modified from the California Health Interview Survey (CHIS) – Social Support Measure |
| **HIV Vulnerabilities** |  |
| Positive STI Test (Lifetime) | Categorical; four categories: ‘Yes, ‘No,’ ‘Never tested,’ or ‘Prefer not to answer.’ Based on the question, “Have you ever tested positive for a sexually transmitted infection (STI)?” |
| Positive STI Test (Past 3 months) | Categorical; four categories: ‘Yes, ‘No,’ ‘Not tested last 3 months,’ or ‘Prefer not to answer.’ Based on the question, “Have you tested positive for a sexually transmitted infection (STI) in the last 3 months?” |
| Sex Work (Lifetime) | Categorical; three categories: ‘Yes,’ ‘No,’ or ‘Prefer not to answer.’ Based on the question, “In your lifetime, have you ever had sex with someone so that they would give you money, drugs, alcohol, food, a place to sleep, or other material goods? By sex, we mean oral, anal, and/or vaginal sex.” |
| Sex Work (Current) | Categorical; three categories: ‘Yes,’ ‘No,’ or ‘Prefer not to answer.’ Based on the question, “In the last 3 months, have you had sex with someone so that they would give you money, drugs, alcohol, food, a place to sleep, or other material goods? By sex, we mean oral, anal, and/or vaginal sex.” |
| HIV Risk (Self-Assessment) | Categorical; five categories: ‘Med to High Risk,’ ‘Low Risk,’ ‘No Low Risk,’ ‘Prefer not to answer,’ or ‘Did not say Yes to HIV Testing.’ Based on the question, “How high do you think your risk for HIV infection is?” |
| No. of Sexual Partners (Past 3 months) | Categorical; four categories: ‘Zero partners,’ ‘One partner,’ ‘2-4 partners,’ or ‘5 + partners.’ Based on the question, “In the last 3 months, how many (different) sexual partners did you have? Please give your best estimate. (If you have not had any sex partners in the past 3 months, please type "0").” |
| Gender of Sex Partners (Past 12 months) | Categorical; four categories: Cisgender men, Cisgender women, Transmen/NB [FAB], or Transwoman/NB [MAB], Based on the question “In the past 12 months, what was/were the genders of your sex partner(s)?” |
| *Note.* HS=High School, GED=General Educational Development, FPL=Federal Poverty Line, NB=Non-Binary, FAB=Female at Birth, MAB=Male at Birth. | |

| **Table S2. Goodness-of-Fit Criteria for Competing Latent Class Models Among Transgender Women in the LITE Study, Eastern and Southern U.S. (N=958)** | | | | | | | | |
| --- | --- | --- | --- | --- | --- | --- | --- | --- |
| Classes | G^2^ | df | Log-Likelihood | AIC | BIC | BLTR | Entropy *R*^2^ | Solution % |
| *Site Based Arm (N = 577)* | | | | | | | | |
| 1 | 1030.46 | 1013 | -3231.36 | 1050.46 | 1094.04 | -- | 1.00 | 100 |
| 2 | 646.29 | 1002 | -3039.28 | 688.29 | 779.81 | 0.001 | 0.66 | 100 |
| 3 | 474.04 | 991 | -2953.15 | 538.04 | 677.49 | 0.001 | 0.81 | 77 |
| **4** | **416.52** | **980** | **-2924.39** | **502.52** | **689.90** | **0.001** | **0.77** | **92** |
| 5 | 376.27 | 969 | -2904.26 | 484.27 | 719.59 | 0.001 | 0.77 | 54 |
| *Online Arm (N = 381)* | | | | | | | | |
| 1 | 611.63 | 1013 | -2001.36 | 631.63 | 671.06 | -- | 1.00 | 100 |
| 2 | 396.49 | 1002 | -1893.79 | 438.49 | 521.29 | 0.001 | 0.75 | 100 |
| 3 | 348.14 | 991 | -1869.61 | 412.14 | 538.31 | 0.001 | 0.72 | 26 |
| **4** | **302.78** | **980** | **-1846.93** | **388.78** | **558.32** | **0.001** | **0.74** | **95** |
| 5 | 267.02 | 969 | -1829.05 | 375.02 | 587.93 | 0.001 | 0.74 | 81 |
| *Note.* AIC = Akaike’s Information Criterion; BIC = Bayesian Information Criterion; BLRT = Bootstrap Likelihood Ratio Test. Solution % is the percentage of times solution was selected out of 100 random sets of starting values. | | | | | | | | |

| **Table S3. Gender-Affirming Characteristics of Sexually Active Transgender Women in the LITE Study, Eastern and Southern U.S. (N=958)** | | | | |
| --- | --- | --- | --- | --- |
| Characteristics | | Site-based (N=577)  n (%) | Online (N=381)  n (%) | Total (N=958)  n (%) |
| **Gender Identity** | Woman, Female | 204 (35) | 74 (19) | 278 (29) |
|  | Transwoman, Transfemale | 268 (46) | 216 (57) | 484 (51) |
|  | Transfeminine, Transfemme | 36 (6) | 45 (12) | 81 (8) |
|  | Non-Binary^†^ | 27 (5) | 28 (7) | 55 (6) |
|  | Woman of trans experience | 26 (5) | 12 (3) | 38 (4) |
|  | Person of trans experience | 3 (0) | 1 (0) | 4 (0) |
|  | Transgender women-Two Spirit | 10 (2) | 3 (1) | 13 (1) |
|  | Other Identity | 3 (1) | 2 (1) | 5 (1) |
| **Hormone Therapy**  **(Past 3 months)** | Yes | 481 (83) | 279 (73) | 760 (79) |
|  | No | 93 (16) | 98 (26) | 191 (20) |
|  | Prefer not to answer | 3 (1) | 4 (1) | 7 (1) |
| **Any Gender**  **Affirming**  **Procedure**^‡§^ | Yes | 299 (52) | 152 (40) | 451 (47) |
| **Trans-specific**  **HIV Prev. Info.**  **(Past 3 months)** | Yes | 156 (27) | 62 (16) | 218 (23) |
|  | No | 395 (68) | 302 (79) | 697 (72) |
|  | Prefer not to answer | 5 (1) | 2 (1) | 7 (1) |
|  | Don’t know | 21 (4) | 15 (4) | 36 (4) |
| **Social Support**  **(Past 6 months)** | High >12 | 261 (45) | 181 (48) | 442 (46) |
|  | Low <=12 | 299 (52) | 192 (50) | 491 (51) |
|  | Prefer not to answer | 17 (3) | 7 (2) | 24 (3) |

*Note.* ^†^Nonbinary category includes agender, gender fluid, gender variant, and gender non-conforming. ^‡^Hair removal/electrolysis, breast augmentation/surgery, orchiectomy/removal of testes, vaginoplasty/bottom surgery, or facial feminization surgery (such as nose, brow, chin, cheeks). ^§^Recoded as Yes=Have had it vs. Do not want it, Not sure if want it, Want it, and Prefer not to answer. Social support is missing data from 1 participant.

| **Table S4. HIV Vulnerabilities of Sexually Active Transgender Women in the LITE Study, Eastern and Southern U.S. (N=958)** | | | | |
| --- | --- | --- | --- | --- |
| Characteristics | | Site-based (N=577)  n (%) | Online (N=381)  n (%) | Total (N=958)  n (%) |
| **Positive STI Test** | Yes | 189 (33) | 42 (11) | 231 (24) |
| **(Lifetime)** | No | 296 (51) | 209 (55) | 505 (53) |
|  | Never tested | 81 (14) | 128 (33) | 209 (22) |
|  | Prefer not to answer | 11 (2) | 2 (1) | 13 (1) |
| **Positive STI Test** | Yes | 34 (6) | 9 (2) | 43 (4) |
| **(Past 3 months)** | No | 209 (36) | 117 (31) | 326 (34) |
|  | Not tested last 3 months | 322 (56) | 252 (66) | 574 (60) |
|  | Prefer not to answer | 12 (2) | 3 (1) | 15 (2) |
| **Sex Work** | Yes | 286 (50) | 112 (29) | 398 (42) |
| **(Lifetime)** | No | 283 (49) | 263 (69) | 546 (57) |
|  | Prefer not to answer | 8 (1) | 6 (2) | 14 (1) |
| **Sex Work** | Yes | 120 (21) | 39 (10) | 159 (17) |
| **(Current)** | No | 447 (77) | 333 (87) | 780 (81) |
|  | Prefer not to answer | 10 (2) | 9 (2) | 19 (2) |
| **HIV Risk** | Med to High Risk | 184 (32) | 74 (19) | 258 (27) |
| **(Self-Assessment)^†^** | Low Risk | 244 (42) | 123 (32) | 367 (38) |
|  | No Risk | 78 (14) | 60 (16) | 138 (15) |
|  | Prefer not to answer | 8 (1) | 1 (1) | 9 (1) |
|  | Did not say Yes to HIV Testing | 62 (11) | 123 (32) | 185 (19) |
| **No. of Sexual** | Zero partners | 53 (9) | 18 (5) | 71 (7) |
| **Partners** | One partner | 175 (30) | 176 (46) | 351 (37) |
| **(Past 3 months)** | 2-4 partners | 195 (34) | 117 (31) | 312 (33) |
|  | 5 + partners | 154 (27) | 70 (18) | 224 (23) |
| **Gender of Sex** | Cisgender men (Yes) | 448 (78) | 165 (43) | 613 (64) |
| **Partners** | No | 125 (22) | 215 (56) | 340 (35) |
| **(Past 12 months)** | Prefer not to answer | 4 (0) | 1 (1) | 5 (1) |
|  | Cisgender women (Yes) | 141 (24) | 186 (49) | 327 (34) |
|  | No | 434 (75) | 194 (51) | 628 (66) |
|  | Prefer not to answer | 2 (0) | 1 (0) | 3 (0) |
|  | Transmen/NB [FAB] (Yes) | 100 (17) | 95 (25) | 199 (21) |
|  | No | 474 (82) | 282 (74) | 756 (79) |
|  | Prefer not to answer | 3 (1) | 4 (1) | 7 (1) |
|  | Transwoman/NB [MAB] (Yes) | 144 (25) | 137 (36) | 283 (30) |
|  | No | 430 (74) | 243 (64) | 673 (70) |
|  | Prefer not to answer | 3 (1) | 1 (0) | 4 (1) |

*Note.* ^†^ Only asked those who have been previously tested for HIV. HIV Risk is missing data from 1 participant. NB=Non-Binary, FAB=Female at Birth, MAB=Male at Birth.

| **Table S5. Item-response Probabilities for Four-Class Model: Probability of Endorsing Each HIV Awareness and Prevention Item-Site Based Among Transgender Women in the LITE Study, Eastern and Southern U.S. (N = 577)** | | | | | |
| --- | --- | --- | --- | --- | --- |
|  |  | Class | | | |
|  |  | 1 | 2 | 3 | 4 |
|  | ***Prevalence in the Sample (%)*** | Limited Strategies and Less Sexually Active | Limited Strategies and Insertive Sex | Limited Strategies and Receptive Sex | Multiple Strategies and Insertive/Receptive Sex |
| **Class Distribution** |  | 15% | 16% | 33% | 36% |
| HIV Info from Orgs (Past 3 months) | 54 | 0.48 | 0.24 | 0.47 | **0.75** |
| HIV Knowledge | 70 | **0.63** | **0.78** | **0.63** | **0.75** |
| PrEP/PEP Awareness | 88 | **0.78** | **0.79** | **0.86** | **0.99** |
| HIV Test  (Past year) | 77 | **0.69** | 0.49 | **0.72** | **0.96** |
| PrEP Use Ever | 33 | 0.20 | 0.01 | 0.04 | **0.80** |
| PEP Use Ever | 18 | 0.11 | 0.00 | 0.02 | 0.44 |
| Condomless Sex  (Past year) | 77 | **0.64** | **0.71** | **0.74** | **0.87** |
| Receptive Anal/Vaginal Sex (Past 3 months) | 70 | 0.13 | 0.29 | **0.91** | **0.93** |
| Insertive Anal/ Vaginal Sex (Past 3 months) | 42 | 0.00 | **0.90** | 0.27 | **0.51** |
| Oral Sex  (Past 3 months) | 84 | 0.01 | **0.95** | **0.99** | **0.97** |
| *Note.* Prevalence is based on N endorsing each variable. Item-response probabilities > .50 appear in bold to facilitate interpretation. | | | | | |

| **Table S6. Item-response Probabilities for Four-Class Model: Probability of Endorsing Each HIV Awareness and Prevention Item-Online Among Transgender Women in the LITE Study, Eastern and Southern U.S. (N = 381)** | | | | | |
| --- | --- | --- | --- | --- | --- |
|  |  | Class | | | |
|  |  | 1 | 2 | 3 | 4 |
|  | ***Prevalence***  ***in the Sample (%)*** | Limited Strategies and Less Sexually Active | Limited Strategies and Insertive Sex | Limited  Strategies and Receptive Sex | Multiple Strategies and Insertive/Receptive Sex |
|  |  |  |  |  |  |
| **Class Distribution** |  | 9% | 36% | 37% | 18% |
| HIV Info from Orgs (Past 3 months) | 45 | 0.36 | 0.26 | 0.44 | **0.90** |
| HIV Knowledge | 65 | **0.59** | **0.59** | **0.68** | **0.73** |
| PrEP/PEP Awareness | 74 | 0.49 | **0.63** | **0.77** | **0.99** |
| HIV Test  (Past year) | 48 | 0.20 | 0.37 | 0.43 | **0.96** |
| PrEP Use Ever | 09 | 0.00 | 0.00 | 0.00 | **0.52** |
| PEP Use Ever | 05 | 0.00 | 0.00 | 0.00 | 0.26 |
| Condomless Sex  (Past year) | 80 | **0.79** | **0.83** | **0.78** | **0.79** |
| Receptive Anal/Vaginal Sex (Past 3 months) | 56 | 0.14 | 0.13 | **0.98** | **0.74** |
| Insertive Anal/ Vaginal Sex (Past 3 months) | 60 | 0.26 | **0.89** | 0.45 | **0.49** |
| Oral Sex  (Past 3 months) | 92 | 0.17 | **0.99** | **0.99** | **0.98** |
| *Note.* Prevalence is based on N endorsing each variable. Item-response probabilities > .50 appear in bold to facilitate interpretation. | | | | | |
